# Accounting for regional background and population size in the detection of spatial clusters and outliers using geostatistical filtering and spatial neutral models: the case of lung cancer in Long Island, New York

**DOI:** 10.1186/1476-072X-3-14

**Published:** 2004-07-23

**Authors:** Pierre Goovaerts, Geoffrey M Jacquez

**Affiliations:** 1 Biomedware, Inc., Ann Arbor, MI, USA

## Abstract

**Background:**

Complete Spatial Randomness (CSR) is the null hypothesis employed by many statistical tests for spatial pattern, such as local cluster or boundary analysis. CSR is however not a relevant null hypothesis for highly complex and organized systems such as those encountered in the environmental and health sciences in which underlying spatial pattern is present. This paper presents a geostatistical approach to filter the noise caused by spatially varying population size and to generate spatially correlated neutral models that account for regional background obtained by geostatistical smoothing of observed mortality rates. These neutral models were used in conjunction with the local Moran statistics to identify spatial clusters and outliers in the geographical distribution of male and female lung cancer in Nassau, Queens, and Suffolk counties, New York, USA.

**Results:**

We developed a typology of neutral models that progressively relaxes the assumptions of null hypotheses, allowing for the presence of spatial autocorrelation, non-uniform risk, and incorporation of spatially heterogeneous population sizes. Incorporation of spatial autocorrelation led to fewer significant ZIP codes than found in previous studies, confirming earlier claims that CSR can lead to over-identification of the number of significant spatial clusters or outliers. Accounting for population size through geostatistical filtering increased the size of clusters while removing most of the spatial outliers. Integration of regional background into the neutral models yielded substantially different spatial clusters and outliers, leading to the identification of ZIP codes where SMR values significantly depart from their regional background.

**Conclusion:**

The approach presented in this paper enables researchers to assess geographic relationships using appropriate null hypotheses that account for the background variation extant in real-world systems. In particular, this new methodology allows one to identify geographic pattern *above and beyond *background variation. The implementation of this approach in spatial statistical software will facilitate the detection of spatial disparities in mortality rates, establishing the rationale for targeted cancer control interventions, including consideration of health services needs, and resource allocation for screening and diagnostic testing. It will allow researchers to systematically evaluate how sensitive their results are to assumptions implicit under alternative null hypotheses.

## Background

Cancer mortality maps are important tools in health research, allowing the identification of spatial patterns, clusters and disease 'hot spots' that often stimulate research to elucidate causative relationships [1,2]. In most spatial analysis software a statistical pattern recognition approach has been implemented whereby a statistic (*e.g. *spatial cluster statistic, autocorrelation statistic, *etc*.) quantifying a relevant aspect of spatial pattern is calculated. The value of this statistic is then compared to the distribution of that statistic's value under a null spatial model. This provides a probabilistic assessment of how unlikely an observed spatial pattern is under the null hypothesis [3]. Waller and Jacquez [4] formalized this approach by identifying five components of a test for spatial pattern.

1. The *test statistic *quantifies a relevant aspect of spatial pattern (*e.g*. Moran's *I*, Geary's *c*, LISA, a spatial clustering metric, *etc*.)

2. The *alternative hypothesis *describes the spatial pattern that the test is designed to detect. This may be a specific alternative, such as clustering near a focus, or it may be the omnibus "not the null hypothesis".

3. The *null hypothesis *describes the spatial pattern expected when the alternative hypothesis is false (*e.g*. Complete Spatial Randomness, often called CSR).

4. The *null spatial model *is a mechanism for generating the reference distribution. This may be based on distribution theory, or it may use randomization (*e.g*. Monte Carlo) techniques.

5. The *reference distribution *is the distribution of the test statistic when the null hypothesis is true.

CSR is the null hypothesis employed by most, if not all, statistical tests for spatial pattern, and is the workhorse of almost all spatial statistical software. Examples of statistics used in these tests include spatial autocorrelation (*e.g*. Moran's *I *and Geary's c); its local counterpart (*e.g*. LISA); geographic boundary statistics (*e.g*. boundary count and mean length), and a host of techniques for identifying hot spots, cold spots and foci. While CSR is useful in some situations, it often is not a relevant null hypothesis for highly complex and organized systems such as those encountered in the environmental and health sciences [5,6]. For such fields CSR may be not relevant because spatial randomness rarely, if ever, occurs – some spatial pattern is almost *always *present. Hence in many situations rejecting CSR has little scientific value because CSR does not describe any plausible state of the system. As emphasized by Ord and Getis [7], Type I errors may abound when statistical tests are applied without regard to the global autocorrelation structure. For example, locations would be identified as hot spots simply because they lie in areas of generally high (or low) values, which would lead one to blend together local peaks and clusters of high (low) values. Even when health professionals are interested in identifying areas with generally high (or low) disease rates, it is still important to account for spatial autocorrelation to avoid an over-identification of the number of significant spatial clusters or outliers. In summary what are needed are realistic models that incorporate background pattern – the spatial and multivariate structure found when the null hypothesis is true.

The term "*Neutral Model*" captures the notion of a plausible system state that can be used as a reasonable null hypothesis (*e.g*. "background variation"). The problem then is to identify spatial patterns *above and beyond *that incorporated into the neutral model, enabling, for example, the detection of cancer clusters beyond background or regional variation in the risk of developing cancer. Neutral models can be generated using simulation techniques developed in the field of geostatistics [8] which provides a set of statistical tools for analyzing and mapping data distributed in space and time. In particular, sequential Gaussian simulation (SGS) allows one to generate realizations of the spatial distribution of rates that reproduce the sample histogram and spatial patterns displayed by the data, and also account for any auxiliary data or information on the local trend [9].

The objective of this paper is to present geostatistical approaches to generating neutral models that account for the spatial dependence of cancer rates, their regional background and spatially heterogeneous population sizes. These models are then used for the detection of local clusters and anomalies in cancer rates. The new methodology is applied to the analysis of the geographical distribution of lung cancer in three counties of Long Island, New York, which have been investigated under the CSR hypothesis in a previous issue of this journal [10,11]

## Methods

### Data

The use of neutral models in cluster analysis will be illustrated using the lung cancer data analysed in [10,11]. This section briefly summarizes the salient features of this dataset, and readers are referred to the above papers for a detailed description.

The New York State Department of Health (NYSDOH) published the cancer incidence data online as part of their Cancer Surveillance Improvement Initiative, . Data have been released on the following four cancers: breast (female only), colorectal (female and male), lung (female and male), and prostate. These data represent newly diagnosed cancer cases in the period 1993–7 assigned to the patient's residence at diagnosis, and they are calculated as the number of cancers for each 100,000 people in the population.

To protect patient privacy, the NYSDOH data provided case counts referenced to ZIP codes rather than individual residences. While ZIP codes are somewhat arbitrary spatial units of analysis with respect to potential health and environmental factors, they provide a convenient way to group the population and preserve confidentiality. The methods presented here do not depend on the spatial unit of aggregation and the reader may use census geography if that is their preference. As in the earlier analysis [10,11], the focus of this study is on the 214 ZIP codes within Nassau, Queens and Suffolk County on Long Island.

Because cancer incidence is related to age, NYSDOH calculated the expected cancer incidence for each ZIP code using the ZIP code's age structure and the average incidence by age class for New York State (direct adjustment). We thus are using an external standard (the state average) rather than an internal standard (the average for Long Island), to calculate the expected incidence. A standardized morbidity ratio (SMR) has been calculated by dividing the observed value by the age-adjusted expected incidence. An SMR value of 1.0 indicates that the observed incidence is the same as expected, lower than 1.0 indicates that fewer than expected cases of cancer occurred, and greater than 1.0 indicates that more than expected occurred.

### Local cluster analysis under CSR

Jacquez and Greiling [10] identified significant clustering and spatial outliers in SMR using Anselin's local Moran test [12] in the ClusterSeer™ Software . The local Moran test evaluates local clustering or spatial autocorrelation by computing the contribution of each location to the Moran's I statistics for the whole study area. Its null hypothesis is that there is no association between SMR values in neighboring ZIP codes. The working (alternative) hypothesis is that spatial clustering exists. For each ZIP code, referenced geographically by its centroid with the vector of spatial coordinates **u **= (x, y), the LISA (Local Indicator of Spatial Autocorrelation) statistic is computed as:



where z(**u**) is the SMR for the ZIP code being tested, which is referred to as the "kernel" hereafter. z(**u**_j_) are the values for the J(**u**) neighboring ZIP codes that are here defined as units sharing a common border or vertex with the kernel **u **(1-st order queen adjacencies). All values are standardized using the mean *m *and standard deviation *s *of the SMR data (here 214 values). Since the standardized values have zero mean, a negative value for the LISA statistics indicates a spatial outlier where the kernel value is much lower or much higher than the surrounding values (e.g. SMR is below the global zero mean while the neighborhood average is above the global zero mean, or conversely). Cluster of low or high values will lead to positive values of the LISA statistics (e.g. both kernel and neighborhood averages are jointly above zero or below zero).

In addition to the sign of the LISA statistics, its magnitude informs on the extent to which kernel and neighborhood values differ. To test whether this difference is significant or not, a Monte Carlo simulation is conducted, which traditionally consists of sampling randomly and without replacement the global distribution of observed rates (i.e. sample histogram), then computing the corresponding simulated neighborhood averages. This operation is repeated many times (e.g. *L *= 999 draws) and these simulated values are multiplied by the kernel value to produce a set of *L *simulated values of the LISA statistics at location **u**:



with z^(l)^(**u**_j_) = F^-1^[p^(l)^(**u**_j_)], F[.] is the sample cumulative distribution function (cdf), and p^(l)^(**u**_j_) is a random number uniformly distributed within 0 and 1. This set represents a numerical approximation of the probability distribution of the LISA statistics at **u**, under the assumption of spatial independence. The observed LISA statistics, LISA(**u**), can then be compared to the probability distribution, allowing the computation of the probability of not rejecting the null hypothesis (so-called *p*-value). Following Jacquez and Greiling [10], we used an adjusted significance level *α *= 0.01101 to account for the fact that the multiple tests (i.e. 214 in this study) are not independent since near ZIP codes share similar neighbors. This significance level was obtained using the Bonferroni adjustment which amounts at dividing the significance level *α *= 0.05 by the average number of neighbors in each test. Thus, every ZIP code where the p-value is lower than 0.01101 will be classified as a significant spatial outlier (HL: high value surrounded by low values, and LH: low value surrounded by high values) or cluster (HH: high value surrounded by high values, and LL: low value surrounded by low values).

### A typology of neutral models

The use of CSR as the null hypothesis means that the distribution of cancer rates is assumed to be spatially random (no autocorrelation) with uniform risk over the study area. In most cases, however, mortality rates are spatially correlated while the risk of developing cancer varies regionally as a result of changes in environmental exposure or other demographic, social, and economic factors. Another weakness of the above test is that it does not consider whether ratio data are based on many or a few cases, thereby ignoring the instability of rates computed from small population sizes. The basic idea of the proposed approach is to generate neutral models that are more realistic in the sense that they incorporate presence of spatial autocorrelation, non-uniform risk, and account for spatially heterogeneous population sizes.

Table 1 provides a typology of neutral models that could be used for inference regarding numerator and denominator, including incidence and prevalence, as well as mortality rates. Model I corresponds to the CSR case, while model II reproduces the spatial correlation of the cancer rates. Model III reflects the situation where environmental exposures or other factors make the risk non-uniform. Models IV through VI allow one to account for the impact of population size on the stability of observed rates. Unlike Model I these more complex neutral models can not be generated simply by shuffling randomly the SMR data across the 214 ZIP codes, and geostatistical simulation techniques to generate each type of model are described below.

**Table 1:** Typology of neutral models. Models differ according to the reproduction of spatial correlation, the incorporation of non-uniform risk, and the filtering of noise caused by spatially varying population sizes.

Risk	Accounting for Population size	
	No	Yes

Uniform, spatially random	**I**	**IV**
Uniform, spatially correlated	**II**	**V**
Heterogeneous, spatially correlated	**III**	**VI**

### Normal score transform of SMR data

The simulation techniques used in this paper assume a multiGaussian distribution for the variable under study, which requires a prior normal score transform of SMR data to ensure that at least their univariate distribution (histogram) is normal. Normal score transform is a graphical transform that allows one to normalize any distribution, regardless of its shape. It can be seen as a correspondence table between equal *p*-quantiles z_p _and y_p _of the z-cdf F(z) (cumulative histogram) and the standard Gaussian cdf G(y). In practice, the normal score transform proceeds in three steps:

1. The N original data z(**u**_*α*_) (i.e. SMR data) are first ranked in ascending order. Since the normal score transform must be monotonic, ties in *z*-values must be broken, which has been done randomly as implemented in GSLIB software [13].

2. The sample cumulative frequency of the datum z(**u**_*α*_) with rank k is then computed as  = k/N - 0.5/N.

3. The normal score transform of the *z*-datum with rank *k *is matched to the -quantile of the standard normal cdf:

*y*(**u**_*α*_) = *φ*(*z*(**u**_*α*_)) = *G*^-1^[*F*(*z*(**u**_*α*_))] = *G*^-1^[]

### Local cluster analysis under spatial neutral model (Model II)

Model II aims to reproduce the pattern of spatial correlation displayed by the data that is here quantified using the normal score semivariogram [8,14] which plots the average squared difference between normal score transformed SMR data as a function of the separation distance and direction between ZIP codes:



Here |**h**| corresponds to the Euclidian distance between two ZIP codes. Note the following discussion can be readily generalized to other distance measures that could be more appropriate to capture contiguity of entities of complex shape: our methodology is general and does not depend on a particular formulation of the distance measures. Following previous simulation studies [9] and in order to account for the noise induced by small population sizes, each pair has been assigned a weight proportional to the square root of the population size, , where n(**u**_*α*_) is the size of the population at risk in the ZIP code with centroid **u**_*α*_. Following an earlier analysis of the data [10], the population in ZIP codes was estimated using 2000 US census numbers.

Spatial neutral models are generated using Sequential Gaussian Simulation (SGS) which proceeds as follows (see [8] p. 380 for more details):

1. Fit a permissible function [9] to the experimental semivariogram (Equation 3). The modeling was here performed using least-square regression [15]. All semivariogram models were bounded, that is a sill is reached for a given distance referred to as the range of influence. The covariance models were then derived by subtracting the semivariogram model from the sill value.

2. Define a random path (i.e. using a random number generator) visiting each ZIP code location **u**_*α *_only once.

3. At each location **u**_*α *_determine the mean and variance of the Gaussian probability distribution of y-values as:





where y^(l)^(**u**_i_) are normal scores simulated at locations previously visited along the random path and located within a search radius from **u**_*α*_, m_Y _is the stationary mean of the variable Y (which is zero following the normal score transform), and C(**u**_i_-**u**_*α*_) is the covariance function of the normal score variable Y for the separation vector **h**_i*α *_= **u**_i_-**u**_*α*_. *λ*_i _are kriging weights obtained by solving the following system of linear equations (simple kriging, SK):



4. Draw a simulated value from the conditional cumulative distribution function (ccdf) of probability and add it to the data set. In other words, the simulated value at **u**_*α *_is , where p^(l) ^is a random number between 0 and 1.

5. Proceed to the next location along the random path, and repeat the two previous steps.

6. Loop until all *N *locations (i.e. N = 214 here) are simulated.

7. Transform the simulated normal scores {y^(l)^(**u**_*α*_); *α *= 1,..., N} so that the target histogram (in this case the global distribution of observed rates, F[.]) is reproduced: z^(l)^(**u**_*α*_) = F^-1^[p^(l)^(**u**_*α*_)] with p^(l)^(**u**_*α*_) = G[y^(l)^(**u**_*α*_)]

The procedure is repeated using a different random path and set of random numbers to generate another realization. Note that these realizations account for only the histogram and semivariogram model of the SMR data (global conditioning), but they are non-conditional to the SMR data themselves (e.g. location of zones of high or low SMR values).

Once the L sets of N simulated SMR values, {z^(l)^(**u**_*α*_); *α *= 1,..., N} have been generated, Equation (2) is applied to each member of this set to compute the simulated values of the LISA statistics at each location **u**. The simulated LISA values form the empirical distribution of the LISA statistics, allowing the calculation of the p-value for the test of hypothesis.

### Local cluster analysis under a locally constrained spatial neutral model (Model III)

The simulation of neutral model II is conducted using a stationary mean for SMR values, which is unrealistic for situations where environmental exposure or other factors make the risk non-uniform. In this instance the researcher wishes to detect spatial pattern *above and beyond *this non-uniform risk. For example, one might want to detect clusters of melanoma beyond those that are explained by the north-south gradient in solar radiation. Non-uniform risk can easily be accounted for in the simulation procedure by replacing the stationary mean m_*Y *_in Equation 4 by locally varying means m_Y_(**u**_*α*_), that is using the following estimate for the mean and variance of the Gaussian ccdf:





where C_R_(**u**_i_-**u**_*α*_) is the covariance function of the residual normal score variable [Y(**u**_*α*_) - m_Y_(**u**_*α*_)] for the separation vector **h**_i*α *_= **u**_i_-**u**_*α*_, and the kriging weights are obtained by solving the following system of linear equations (simple kriging with local means, SKlm):



The first step in the generation of model III is the computation of the local means m_Y_(**u**_*α*_), which define the reference background risk the user wants to consider for the null hypothesis. In this paper a smooth model of background risk values was obtained by using the following kriging estimator of the local means of observed SMR data:



The kriging weights are calculated in two-steps. First, the following "kriging of the local mean" system [8] is solved:



Then, to incorporate data reliability due to spatially varying population size directly into the geostatistical filter the kriging weights are rescaled, following [9], as:



This rescaling is applied separately to the negative and positive kriging weights, keeping constant the overall contribution of these two sets of weights; that is the sum of positive (negative) kriging weights is the same before and after rescaling, which ensures that the unbiasedness constraint in system (11) is still satisfied. Note that although the population size is incorporated in the estimation of the local means, it is not accounted for directly into the test of hypothesis, which will be achieved using Models IV through VI introduced below.

Once the local means of the normal score transformed SMR data have been estimated, they are subtracted from the SMR values and the semivariogram of residuals is estimated and modelled. Then, the simulation is performed using SGS and SKlm. Last, the L realizations are used to derive the empirical probability distribution of the LISA statistics and the p-value of the test is computed.

### Accounting for population size in local cluster analysis (Models IV to VI)

The neutral models introduced so far ignore the fact that cancer rates estimated over small areas, such as United States ZIP code areas or census tracts, tend to be less reliable [16,17], hence larger fluctuations among simulated rates are expected at these locations. If ignored, large differences in population size decrease the ability of Moran's I to detect true clustering. There are essentially three approaches to incorporate population sizes in cluster detection: 1) randomly shuffle the cases rather than the rates (i.e. under a heterogeneous Poisson model the cases are allocated to each area using hypergeometric sampling [18]), 2) use a modified version of the test statistics (i.e. Oden's I pop [19] or Waldhör's I [20]), and 3) transform or standardize the rates first, then compute the LISA statistics on the results (i.e. Empirical Bayes Index [21], Cressie's transform [14 p.385–402], or any other smoothing algorithm [17,22]). In this paper, the third approach has been adopted and the noise caused by small population sizes was filtered using a variant of the estimator introduced in equation 10:



The kriging weights are calculated in two-steps. First, the following system is solved:



with *g*_0 _= *b*_0 _× (1-*δ*(***u***_*i*_-***u***_*α*_)) where *b*_0 _is the nugget variance in the weighted semivariogram model of SMR data, and *δ*(***u***_*i*_-***u***_*α*_) = *0 *if ***u***_*i *_= ***u***_*α *_and 1 otherwise. Then, to incorporate data reliability (i.e. population size) directly into the geostatistical filter the kriging weights are rescaled according to Equation 12. The ability of the proposed approach to reconstruct the underlying disease risk from observed mortality rates has been tested in extensive simulation studies [9].

## Results and discussion

### Generating spatial neutral models

**Figure 1:**
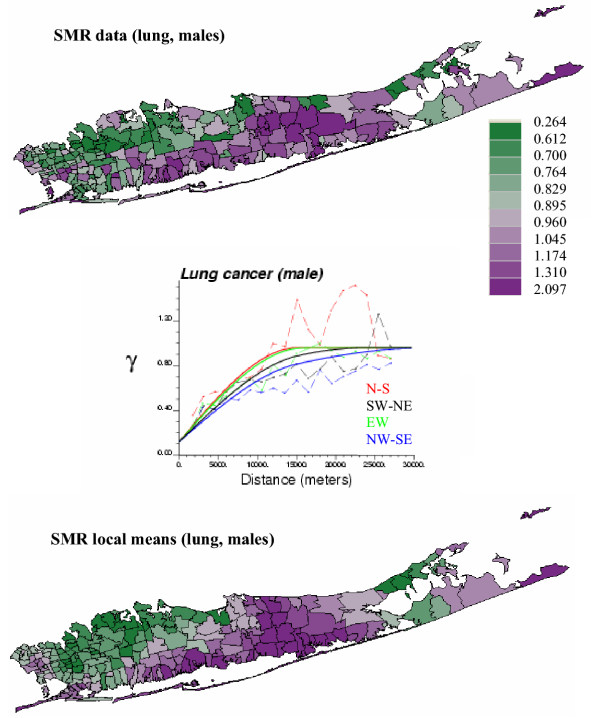
**Geographic distribution and spatial variability of male lung cancer. **The fill color in each ZIP code represents the SMR, with green indicating relatively low SMR and purple representing relatively high SMR (categories correspond to deciles of the histogram of rates). From these rates, a population-weighted semivariogram is computed in four directions. The semivariogram model (solid line) is used to filter the noise and short-range variability of observed SMR, yielding a smooth map of SMR local means (regional background).

**Figure 2:**
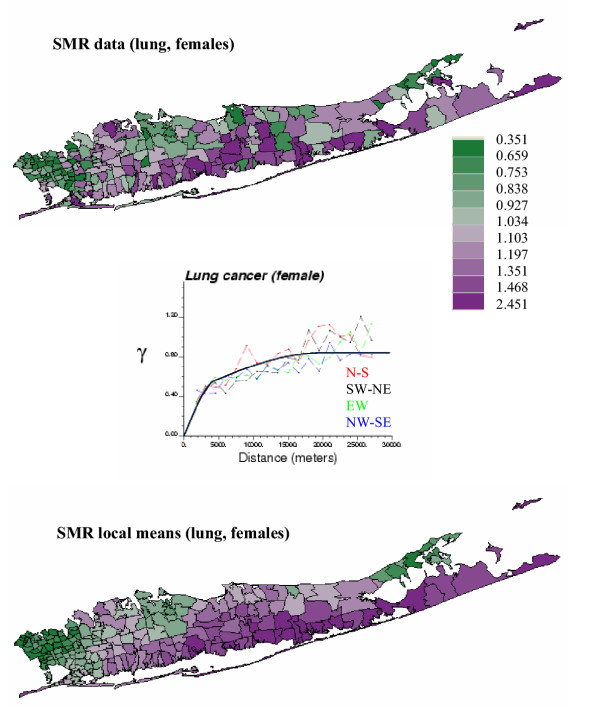
**Geographic distribution and spatial variability of female lung cancer. **The fill color in each ZIP code represents the SMR, with green indicating relatively low SMR and purple representing relatively high SMR (categories correspond to deciles of the histogram of rates). From these rates, a population-weighted semivariogram is computed in four directions. The semivariogram model (solid line) is used to filter the noise and short-range variability of observed SMR, yielding a smooth map of SMR local means (regional background)

**Figure 3:**
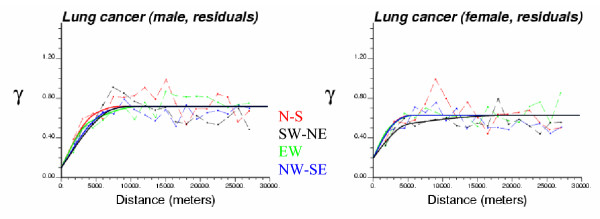
**Residual semivariograms for male and female lung cancer. **The regional background displayed at the bottom of Figures 1 and 2 is subtracted from the maps of SMR data, and the spatial variability of these residuals is characterized by population-weighted semivariograms computed in four directions.

Figures 1 and 2 (top graphs) show the geographic distribution of lung cancer in males and females (aggregated to the ZIP code level), in Long Island, New-York. Middle graphs show the experimental weighted semivariograms computed in four directions from the normal score transforms of SMR data. For both males and females SMR normal scores exhibit a range of autocorrelation of about 15 km, with smaller variability (i.e. lower semivariogram values) observed along the NW-SE direction. The spatial anisotropy is less pronounced for female lung cancer and an isotropic model was fitted (solid black line). Regional background is further revealed once the noise and short-range variability of SMR data has been removed using factorial kriging (Equation 10) and the semivariogram model fitted to sampled values (solid line in middle graphs), see Figures 1 and 2 (bottom graph). High SMR values are recorded mainly along the Southern shore of the Island for both genders, while differences between males and females are more striking for low value: the lowest SMR values are observed in the westernmost part of Long Island for females and slightly more to the east for the males. These maps of regional background were subtracted from the original SMR maps, and the spatial autocorrelation of the corresponding residuals was quantified using the experimental semivariograms displayed in Figure 3. Since some of the spatially correlated variability is captured by the regional background, the residual semivariograms show lower sills and shorter ranges relatively to the SMR semivariograms of Figures 1 and 2.

**Figure 4:**
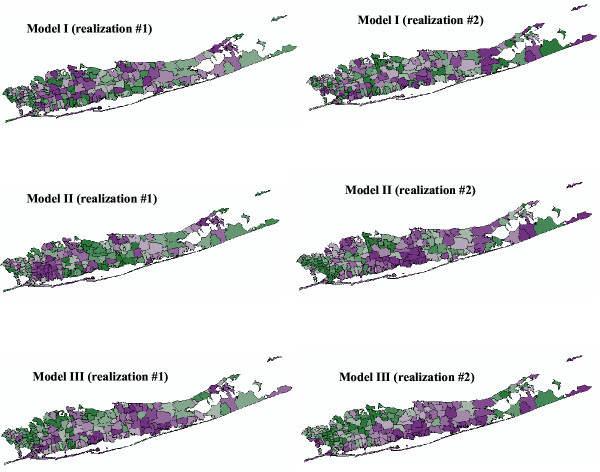
**Different neutral models for male lung cancer. **The fill color in each ZIP code represents the simulated SMR, with green indicating relatively low SMR and purple representing relatively high SMR (categories correspond to deciles of the histogram of simulated rates). Simulated maps (realizations) of the spatial distribution of lung cancer SMR data are generated under the assumption of complete spatial randomness (Model I), or created using geostatistical simulation in order to reproduce the spatial autocorrelation displayed by observed rates (Model II) as well as the regional background, i.e. SMR local means (Model III).

One hundred realizations of neutral Models I through III were generated using Sequential Gaussian simulation and the information displayed in Figures 1 to 3. The first two realizations of each model for male lung cancer are displayed in Figure 4. The two top maps (model I), which were obtained by shuffling randomly the 214 ZIP code SMR data in Figure 1 (top map), illustrate the simplistic nature of CSR as null hypothesis in cluster detection. Spatial patterns are reproduced by the middle maps (Model II) where one notices groups of low and high simulated SMR values the position of which changes from one realization to the next since the simulation is not conditioned locally to the observed rates. The regional background displayed in Figure 1 (bottom graph) is incorporated in Model III, which reduces fluctuations among realizations and led, for example, to high SMR values being consistently simulated in the central part of Long Island.

### Accounting for population size in spatial neutral models

**Figure 5:**
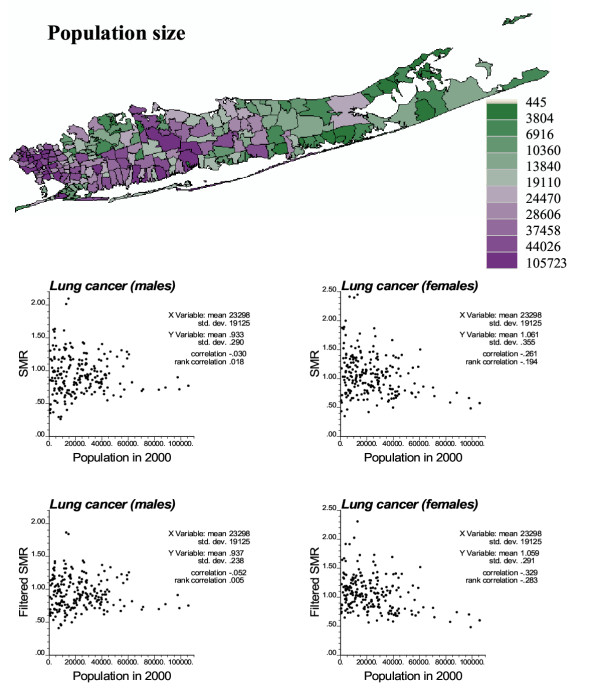
**Geographic distribution of population size (male + female) and its impact on stability of SMR values. **The fill color in each ZIP code represents the 2000 population size, with green indicating sparsely populated ZIP codes and purple representing larger population sizes (categories correspond to deciles of the histogram of sizes). The scatterplots illustrate the larger spread of measured SMR for ZIP codes with low population and how the extreme rates recorded in these ZIP codes are smoothed by geostatistical filtering.

Population in Long Island ZIP codes can vary substantially, ranging from 445 to 105,723, with a mean of 23,298, see Figure 5 (top graph). Population sizes also display a strong spatial pattern, with a gradient from highly populated ZIP codes in the western part of Long Island to the sparsely populated eastern part. The scattergrams in Figure 5 illustrate how the population size impacts the magnitude of fluctuations among SMR data. As the ZIP codes become less populated the variability among SMR values increases, which reflects the smaller reliability of the rates inferred from small populations at risk and makes problematic the later detection of clusters or spatial outliers.

**Figure 6:**
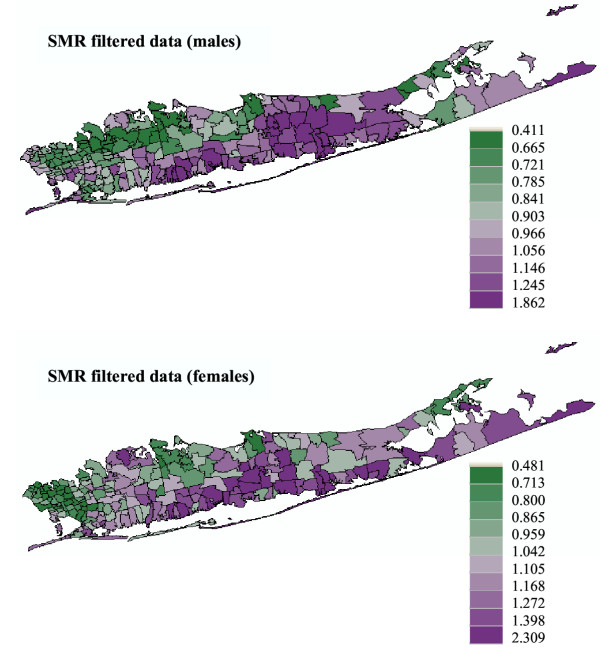
**Geostatistical filtering of male and female lung cancer data. **The fill color in each ZIP code represents the noise-filtered SMR, with green indicating relatively low SMR and purple representing relatively high SMR (categories correspond to deciles of the histogram of filtered rates).

Using factorial kriging and the SMR semivariogram models displayed in Figures 1 and 2, the noise caused by small population sizes was geostatistically filtered from SMR maps: compare filtered maps in Figure 6 with original maps shown at the top of Figures 1 and 2. While the noise filtering does not change the mean of the SMR data, their standard deviation decreases: 0.290 to 0.238 for males and 0.355 to 0.329 for females. The larger decrease observed for male SMR values is caused by the higher amount of noise (i.e. relative nugget effect) reflected as the discontinuity at the origin of the semivariogram. The scattergrams at the bottom of Figure 5 indicate that the geostatistical filtering changes mainly the extreme rates recorded for sparsely populated ZIP codes. Then, one hundred realizations of neutral Model IV through VI were generated using Sequential Gaussian simulation and the filtered SMR maps statistics.

### Local cluster analysis under various neutral models

#### Female

**Figure 7:**
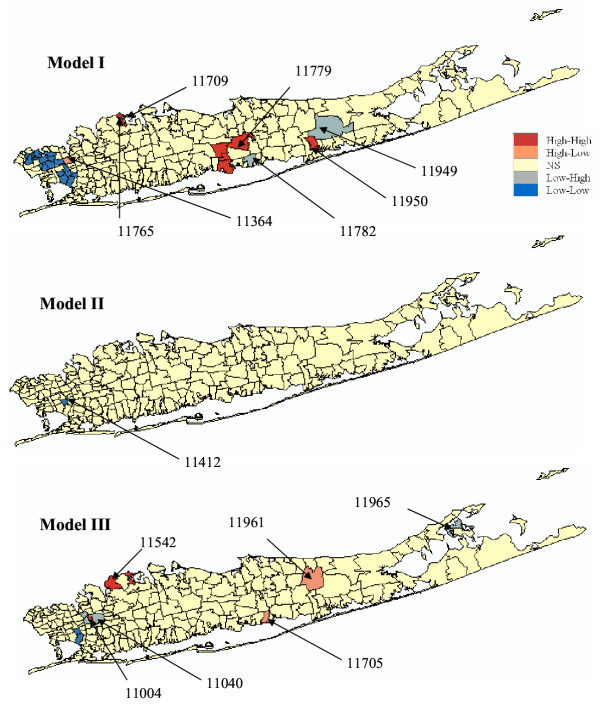
**Results of the local cluster analysis conducted for female lung cancer using neutral models I to III. **The fill color in each ZIP code represents the classification into significant low-low or high-high clusters, as well as high-low or low-high outliers. Yellow indicates ZIP codes that have not been found significant using an adjusted significance level *α *= 0.01101.

**Figure 8:**
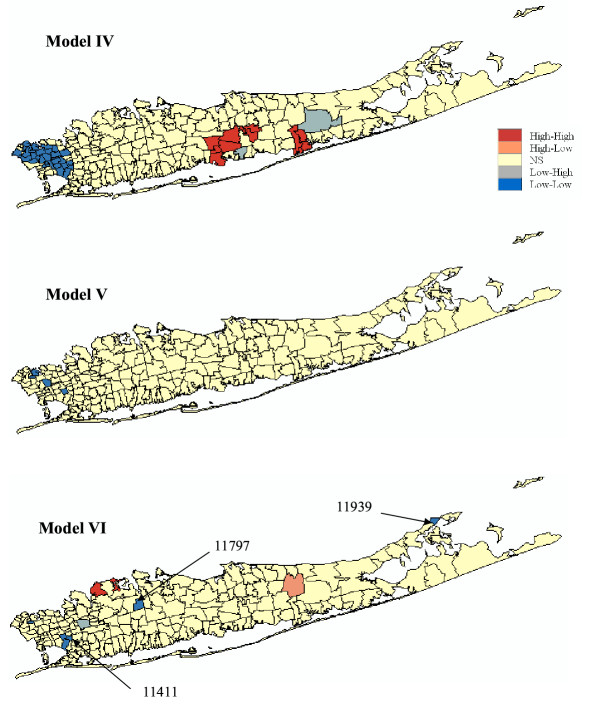
**Results of the local cluster analysis conducted for female lung cancer using neutral models IV to VI. **The fill color in each ZIP code represents the classification into significant low-low or high-high clusters, as well as high-low or low-high outliers. Yellow indicates ZIP codes that have not been found significant using an adjusted significance level *α *= 0.01101.

Figures 7 and 8 show the results of the cluster analysis for female SMR values under the neutral models I through VI, while Table 2 lists the exact number of ZIP codes classified as significant clusters of high values (HH) or low values (LL), and outliers (LH and HL). Table 2 also indicates how the p-value varies among neutral models, highlighting the fact that depending on the assumption being made, the size and locations of clusters/outliers can change.

**Table 2:** Number of significant zip codes for the different types of cluster/outliers and neutral models. Results are reported for female lung cancer. Numbers between parentheses indicate zip codes that have similar classification under the reference Model I (CSR). Summary statistics for the p-values are also provided.

	Neutral Model Type					
	Model I	Model II	Model III	Model IV	Model V	Model VI

High-High	7	0(0)	4(2)	10(5)	0(0)	3(2)
High-Low	1	0(0)	2(0)	0(0)	0(0)	1(0)
Low-High	2	0(0)	4(0)	2(2)	0(0)	1(0)
Low-Low	18	1(0)	2(2)	31(18)	4(4)	6(4)
**P-value**						
Mean	0.178	0.230	0.405	0.166	0.237	0.394
CV	85.9%	62.5%	71.3%	95.3%	62.2%	76.9%

Under the CSR model (Model I), results similar to the ones reported in [11] were found. First, the local Moran test identified a single, large cluster of low SMR extending through portions of Flushing in the north and Jamaica in the south. Next to this cluster is the only high-low outlier: Oakland Gardens (11364) with a SMR of 1.116. Sayville (11782) is a significant spatial outlier with low SMR (72% of the New York average), though its SMR has a wide confidence interval resulting from the small number of observed cases there (15,896 habitants). Thus, while statistically distinct from its neighbors, it does not have significantly reduced risk. This is also the case for the second low-high outlier, Manorville (11949, 11,384 habitants), which has a SMR close to one but is located in the western part of Long Island where background rates are higher. Several local clusters of high SMR values occurred in the more central portions of Long Island. There is a cluster in north-mid Long Island, made up of two significant local clusters centered on Bayville (11709) and Mill Neck (11765). This cluster has about 60–70% higher SMR than the New York state average. A large cluster in south central Long Island is composed of four local clusters centered on Ronkonkama (11779), Central Islip (11722), Islip Terrace (11752), and East Islip (11730). This cluster has an SMR about 40% higher than the New York state average. Further east is a third cluster of high female lung cancer incidence centered on Mastic (11950) and including several adjacent ZIP codes. Its SMR is about 60% higher than the New York state average.

**Figure 9:**
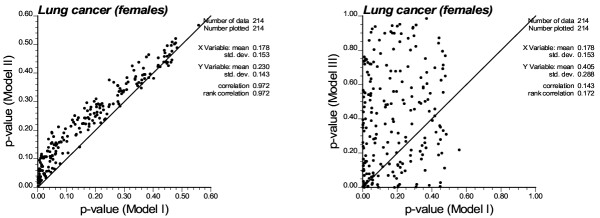
**Scatterplots of the p-values obtained when conducting the local cluster analysis under CSR assumption (Model I) or more complex neutral models. **Model III reproduces the pattern of spatial correlation as well as the regional background of SMR values, while Model II accounts only for the spatial correlation.

**Figure 10:**
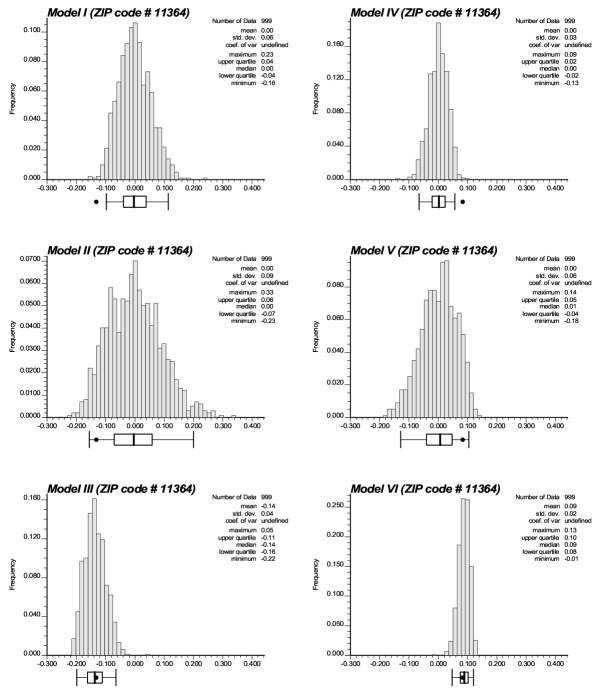
**Histograms of the values of the LISA statistics simulated for ZIP code #11364 (Oakland Gardens) under different neutral models. **The black dot denotes the observed LISA statistics which lies inside the 0.95 probability interval for all models except Models I and IV developed under the CSR assumption.

Accounting for spatial autocorrelation (i.e. Model II) leads to a substantial reduction in the size of significant clusters compared to the CSR assumption. In fact only one ZIP code is a significant low-low cluster under Model II: Saint Albans (11412) which was the center of the Southern low-low cluster detected under CSR. The scattergram in Figure 9 (left graph) shows that the use of spatially correlated neutral models leads to larger p-values on average (0.23 vs. 0.18), and those are highly correlated with the ones obtained under CSR (Model I). These larger p-values cause a substantial reduction in the size of significant ZIP codes, since fewer units exceed the adjusted significance level *α *of 0.01101. The reason for that increase in p-values is illustrated for the ZIP code #11364 (Oakland Gardens) which was the only unit classified as high-low outlier under neutral model I. Figure 10 (left top graph) shows the distribution of simulated values of the LISA statistics for that ZIP code. Clearly, the variance of the distribution is much larger than the results obtained under CSR, while both means are equal to zero. The spatial autocorrelation of simulated rates increases the likelihood that the J neighboring values are jointly small or high, causing the neighborhood average, hence the LISA value, to exhibit much larger fluctuations among realizations. Consequently, the probability that the observed LISA statistics falls in the tails of the simulated distribution decreases, leading to a larger p-value (0.061 versus 0.003) and a ZIP code that is no longer a significant outlier.

The map of significant ZIP codes at the bottom of Figure 7 bears little resemblance with the maps obtained under the neutral models I and II. This is expected since Model III addresses a different question, namely the detection of local departures from the regional background. Thus, in general, one would expect HL and LH outliers to be more frequent than spatial clusters HH or LL. Also the local constraining of the neutral models to the regional background causes less variation among realizations, leading to the J neighboring values being consistently either small or large across the realizations. Thus the distribution of 999 simulated LISA values is expected to be narrower than for the two previous models with a shift in the mean. This is illustrated for the ZIP code #11364 in Figure 10 (left bottom graph). Because this unit is located in a low-valued area, the use of neutral models reproducing the regional background yields smaller simulated LISA values (average = -0.14 instead of 0.0). In high-valued areas, the shift is expected to be in the opposite way, leading to a larger range of p-values observed across the area, see the scattergram in Figure 9 (right graph). Table 2 and Figure 9 indicate that the p-values are of larger magnitude (average: 0.405 versus 0.23 for Model II) and weakly correlated with the ones obtained under CSR.

For female lung cancer, the same numbers of ZIP codes (6) were classified as significant outliers or clusters under neutral model III. The two low-low clusters are Springfield Gardens (11413) and Saint Albans, which was the only significant unit under neutral model II. These ZIP codes are both located in the western part of Long Island with low background SMR values, and in the same area the following three low-high outliers are found: Bellerose (11426), Little Neck (11362), and New Hyde Park (11040) surrounding the high-high cluster Glen Oaks (11004). The last low-high outlier is found in Shelter Island Heights (11965) in the eastern part of Long Island, though its SMR (72% of the New York average) has a wide confidence interval resulting from the small number of observed cases there (1,080 habitants). The two high-low outliers are found in central Long Island characterized by a low SMR background level: Ridge (11961) and Bayport (11705) with SMR values 20 to 40% higher than the New York state average. Three more clusters of high SMR (1.15 to 1.20) are found in the North western part of Long Island, next to the large group of low SMR recorded in Flushing and Jamaica: Bayville (11709), Mill Neck (11765), and Glen Cove (11542).

For all three types of model, accounting for population size through geostatistical filtering leads to a larger number of ZIP codes classified as clusters and fewer outliers, see Figure 8 and Table 2. This result can be explained by the smoothing of local fluctuations, in particular the ones recorded in sparsely populated ZIP codes, yielding larger and more compact clusters, such as for Model IV. Figure 10 (right column) also shows that this smoothing halves the standard deviation of the distributions of simulated LISA statistics. Major differences between Models I and IV include bigger and more compacts clusters of low and high SMR values, the disappearance of two sparsely populated high-high clusters (Bayville and Mill Neck, with 7,134 and 732 habitants, respectively), and the classification of a former high-low cluster into a low-low cluster (Oakland Gardens) since the filtered rate becomes slightly lower than the global mean. A similar trend is observed for spatially correlated neutral models where the filtering increases the number of significant low-low clusters from one to four, all located in the eastern part of Long Island. The comparison of Models III and IV indicates the disappearance of a few sparsely populated ZIP codes which were classified as spatial outliers prior to filtering: HL (Bayport, 8,006 habitants), LH (Shelter Island Heights, Bellerose and Little Neck, with 1,080, 18,726 and 17,502 habitants). The only remaining LH cluster is New Hyde Park which has 39,156 habitants. The HH cluster (Glen Oaks, 14,682 habitants) also disappeared. Conversely, three other ZIP codes with populations ranging from 776 to 21,282 became significant LL clusters under Model IV: East Marion (11939), Woodbury (11797), and Cambria Heights (11411).

Across all six types of neutral models, only one out of 214 ZIP codes is consistently classified into the same category: the low-low cluster at Saint Albans (11412) which has a SMR = 0.82 and a population of 37,452. The stability of this cluster under alternative specifications of the statistical null hypothesis can be used by cancer surveillance and control efforts to quantify the degree of confidence associated with this cancer cluster.

#### Male

**Figure 11:**
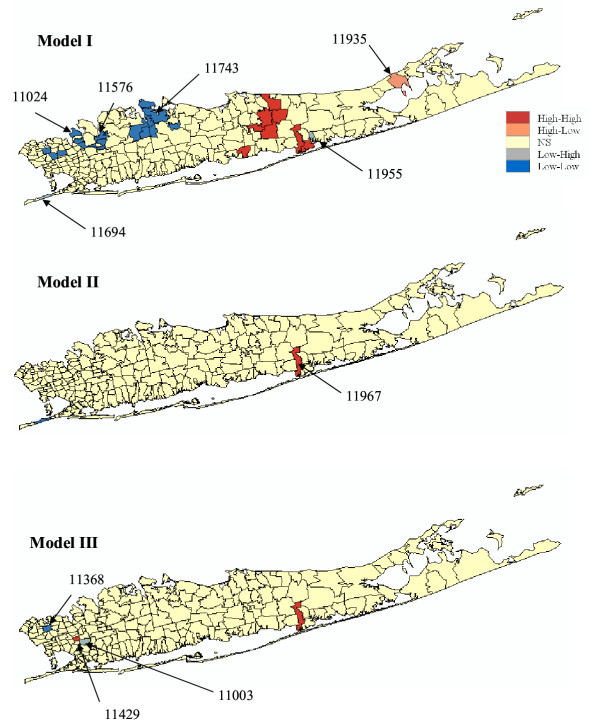
**Results of the local cluster analysis conducted for male lung cancer using neutral models I to III. **The fill color in each ZIP code represents the classification into significant low-low or high-high clusters, as well as high-low or low-high outliers. Yellow indicates ZIP codes that have not been found significant using an adjusted significance level *α *= 0.01101.

**Figure 12:**
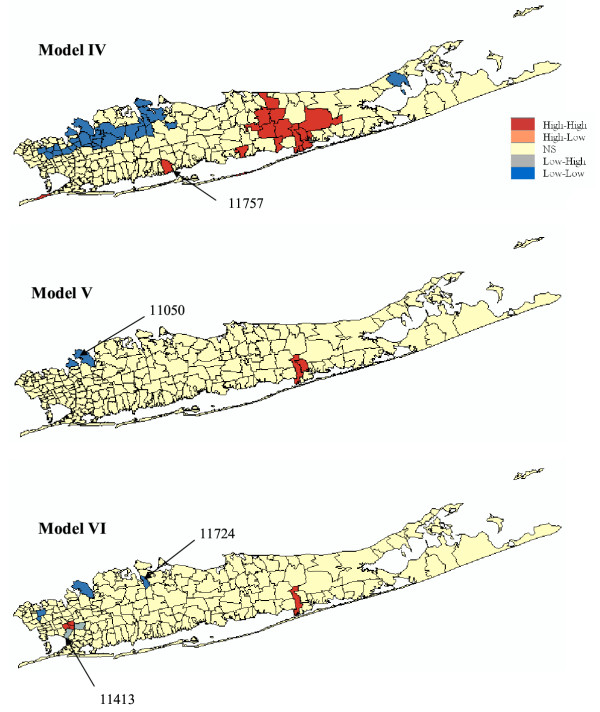
**Results of the local cluster analysis conducted for male lung cancer using neutral models IV to VI. **The fill color in each ZIP code represents the classification into significant low-low or high-high clusters, as well as high-low or low-high outliers. Yellow indicates ZIP codes that have not been found significant using an adjusted significance level *α *= 0.01101.

Results of the cluster analysis for male lung cancer are displayed in Figures 11 and 12 and reported in Table 3. Model I (CSR assumption) yields the same results as the one reported in [11]. Three local clusters of low SMR values were identified, centred on Great Neck (ZIP 11024), Roslyn (11576), and Huntington (11743), all in the northwest portion of Long Island. These clusters are typified by lung cancer SMR values that are 50–75% of the New York State average. A large cluster of lung cancer SMR 20–60% higher than the New York average was identified in central Long Island. Cutchogue (11935) was found a significant high-low outlier although its small population (3,444) impacts the reliability of the morbidity ratio. The two low-high outliers are Moriches (11955) and Rockaway Park (11694).

As for female lung cancer, accounting for spatial autocorrelation (i.e. Model II) leads to fewer significant ZIP codes compared to the common CSR assumption. Only two units are now significant: high-high cluster at Shirley (11967) and a low-high outlier at Rockaway Park (11694). Changes are also substantial when looking at results obtained under neutral model III. We found two high-high clusters: Shirley (11967) and Queens Village (11429), one low-low cluster: Corona (11368), and one low-high outlier: Elmont (11003).

Accounting for population size in the cluster analysis (Model IV) enhances the size and compactness of the two major clusters of low and high SMR values, while the classification of three sparsely sampled ZIP codes (Cutchogue, Moriches and Rockaway Park with 3,444, 2,652 and 19,278 habitants respectively) changed from spatial outliers to clusters. A new cluster of high SMR values (SMR = 1.29) is also found in Lindenhurst (11757). Using spatially correlated neutral models the geostatistical filtering (Model V) reveals a new cluster of low SMR values in Port Washington (11050) and Great Neck (11024) with lung cancer SMR values that are 70% of the New York State average. Comparison of Models III and VI indicates that besides increasing the size of clusters identified under Model II geostatistical filtering leads to the identification of a new low-low cluster: Cold Spring Harbor (11724, with a SMR 63% below the New York state average) and one low-high outlier: Springfield Gardens (11413, SMR = 0.80).

Across all six types of neutral models, only one out of 214 ZIP codes is consistently classified into the same category: the high-high cluster at Shirley (11967) which has a SMR = 1.157 and a population of 24,942.

### How many realizations are needed?

**Figure 13:**
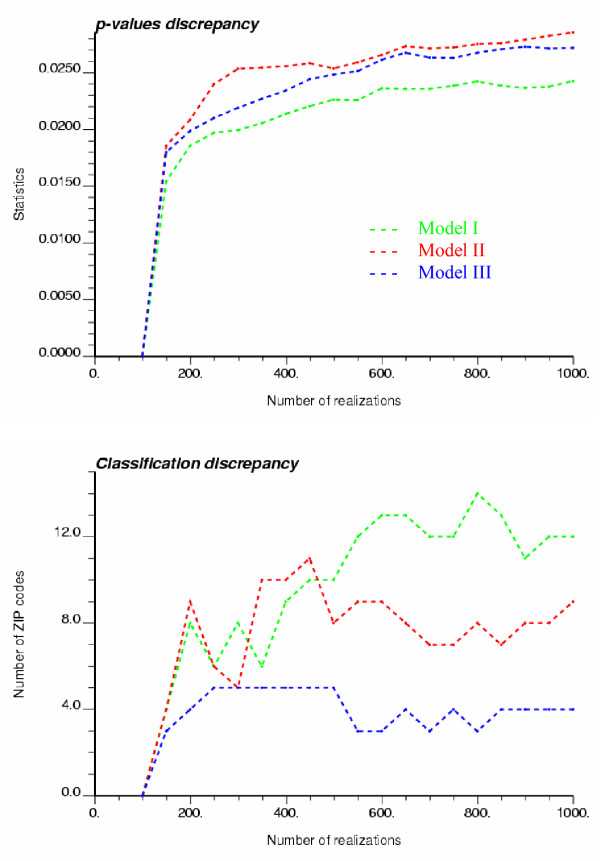
**Impact of the number of realizations and type of neutral models on the stability of local cluster analysis results. **The left graph displays the absolute value of the average change in p-value as the number of realizations increases from 99 to 999. The right graph shows the number of ZIP codes that are classified differently as the number of realizations increases from 99 to 999.

The use of randomization in test of hypothesis relies on the assumption that the space of solution is sampled fairly exhaustively and uniformly (equally-probable realizations [23]). It is thus necessary to investigate how conclusions change as a function of the number of neutral models generated. For example, Figure 13 shows the influence of increasing the sample size from 99 to 999 on the average difference in terms of p-value and classification of ZIP codes into significant outliers and clusters (the reference is the results obtained using 99 realizations). All curves exhibit a plateau within this range of sampling intensity, although the asymptotic behavior depends on the type of neutral models. This result indicates that for this case study enough realizations of neutral models were generated to yield stable classifications of ZIP codes.

## Conclusions

Cancer mortality maps are used by public health officials to identify areas of excess and to guide surveillance and control activities. Maps of incidence as well as mortality are used as input to disease clustering procedures whose purpose is to identify local areas of excess and deficit. While some controversy revolves around the utility of these techniques, it is indisputable that the finding of a confirmed cancer cluster is often of considerable concern. The accurate quantification of local excesses and deficits, as well as regional trends and differences in cancer incidence and mortality, is therefore a problem of considerable practical importance.

Arguably one of the biggest problems facing spatial epidemiology and exposure assessment is that of identifying geographic pattern (*e.g. *hotspots, coldspots, clusters, etc) *above and beyond *background variation. Most, if not all, environmental contaminants and diseases with potential environmental causes occur at a background level in the absence of a pollution- or disease-generating process. Nonetheless, this background pattern is typically ignored in spatial analyses that employ null hypotheses of complete spatial randomness. Because some spatial dependency is expected at background levels, CSR often is an inappropriate null hypothesis.

When should the different types of neutral models be employed? The 6 types of neutral models presented here represent permutations of whether or not population size is accounted for, and 3 types of underlying risk models. As a rule of thumb one should employ that neutral model or those neutral models that most closely correspond to the spatial pattern expected in the absence of the alternative spatial process. So, for a cluster study one would select those neutral models that specify the risk function deemed most likely in the absence of spatial clustering. When working with rates spatial heterogeneity in the size of the at-risk population should always be accounted for, and selections from neutral models of types IV through VI are appropriate. When in doubt about which neutral model to employ, it makes sense to use several in order to determine how sensitive the results are to specification (and misspecification) of the null hypothesis. To the authors' collective knowledge, CSR is rarely if ever encountered in real-world biological systems. It is an apt descriptor of the "snow" that used to appear on late-night television when the programming day was over. It thus seems that neutral model types I and IV will seldom be appropriate. They perhaps will prove most useful for evaluating the extent of bias in past studies that employed CSR.

The approach presented in this paper enables researchers to assess geographic relationships using appropriate null hypotheses that account for the spatial correlation and background variation modeled from the observed rates and any ancillary information (i.e. exposure model). An immediate consequence of using more realistic (i.e. spatially correlated) neutral models are larger p-values, leading to a substantial reduction in the number of ZIP codes declared significant outliers or clusters across Long Island. This result confirms earlier findings that CSR often leads to an over-identification of the number of significant spatial clusters or outliers. These false positives have potentially serious consequences in that it can lead to public alarm and demands for investigation by already stretched state health departments. The drop in the number of significant units is however accentuated by the use of an adjusted significance level (Bonferroni adjustment) to account for the correlation between the tests conducted at neighboring ZIP codes. Further research should investigate the redundancy between the use of spatially correlated neutral models and adjusted significance level, which might lead to an "under-identification" of the number of significant spatial clusters or outliers.

When the constraint of local conditioning of neutral models is superimposed to the reproduction of spatial autocorrelation (i.e. model III), the approach allows one to detect local departures from the conditioning background specified by the user. In this paper, this background was identified to the regional variability of SMR data which was estimated geostatistically. Future research will investigate the use of exposure models for local conditioning of neutral models, leading to the detection of clustered or isolated geographical units that depart significantly from the cancer rates expected from exposure data. A similar approach has recently been implemented whereby the regional background observed in the past has been incorporated into the geostatistical simulation of neutral models [24]. This new methodology allowed one to identify geographic pattern *above and beyond *background variation displayed in prior time intervals for cervix cancer mortality rates.

Another issue, which often impacts the results of cluster analysis, is the lack of reliability of rates inferred from small populations. If ignored, large differences in population size decrease the ability of Moran's I to detect true clustering/departures from spatial randomness. A geostatistical smoother, which accounts for the spatial pattern of SMR data (i.e. anisotropic variability, range of autocorrelation), has been applied to eliminate the random variability that appeared as a nugget effect on the experimental semivariograms. The smoothing of local fluctuations, in particular the ones recorded in sparsely populated ZIP codes, resulted in the detection of larger and more compact clusters of low or high SMR values as well as the disappearance of some unreliable spatial outliers. Geostatistical filters are very flexible and could be used to filter short-range variability in addition to the noise created by small population sizes. In this case, the focus of the analysis would be on the regional background of the data, allowing the detection of regional clusters.

The neutral models and methods in this paper make possible, for the first time ever, evaluation of the sensitivity of the results of cluster or boundary analyses to specification of the null hypothesis. Within a study, this will provide detailed quantification of the reliability of the results, and will identify those areas that are stable (i.e. always classified as a member of a cluster or not) or whose classification is highly sensitive to specification of the null hypothesis. This end result will be a spatially explicit analysis of potential false positives and false negatives.

## Authors' contributions

Authors PG and GMJ collaborated intensely on all aspects of the manuscript, from research design to data preparation. PG carried out most of the geostatistical and local cluster analysis and drafted the manuscript. Both authors read and approved the final manuscript.

**Table 3:** Number of significant zip codes for the different types of cluster/outliers and neutral models. Results are reported for male lung cancer. Numbers between parentheses indicate zip codes that have similar classification under the reference Model I (CSR). Summary statistics for the p-values are also provided.

	Neutral Model Type					
	Model I	Model II	Model III	Model IV	Model V	Model VI

High-High	8	1(1)	2(1)	15(8)	2(1)	4(1)
High-Low	1	0(0)	0(0)	0(0)	0(0)	0(0)
Low-High	2	1(1)	1(0)	1(1)	0(0)	2(0)
Low-Low	14	0(0)	1(1)	24(14)	2(1)	4(2)
**P-value**						
Mean	0.185	0.259	0.368	0.166	0.251	0.371
CV	83.4%	54.8%	71.8%	92.9%	58.9%	80.5%
